# I-ONE therapy in patients undergoing total knee arthroplasty: a prospective, randomized and controlled study

**DOI:** 10.1186/1471-2474-13-88

**Published:** 2012-06-06

**Authors:** Biagio Moretti, Angela Notarnicola, Lorenzo Moretti, Stefania Setti, Francesca De Terlizzi, Vito Pesce, Vittorio Patella

**Affiliations:** 1 Department of Clinical Methodology and Surgical Techniques, Orthopaedics Section, University of Bari, Piazza Giulio Cesare n 11, 70124, Bari, Italy; 2 IGEA SpA - Clinical Biophysics, Via Parmenide 10/A, 41012, Carpi (Mo), Italy

**Keywords:** Total Knee arthroplasty, Pulsed electromagnetic fields, I-ONE therapy, Pain, Inflammation, Functional recovery, Short-term effect, Long-term effect, Knee society score, SF-36 health survey

## Abstract

**Background:**

Total knee arthroplasty (TKA) is often associated with a severe local inflammatory reaction which, unless controlled, leads to persistent pain up to one year after surgery. Standard and accelerated rehabilitation protocols are currently being implemented after TKA, but no consensus exists regarding the long-term effects. Biophysical stimulation with pulsed electromagnetic fields (PEMFs) has been demonstrated to exert an anti-inflammatory effect, to promote early functional recovery and to maintain a positive long-term effect in patients undergoing joint arthroscopy. The aim of this study was to evaluate whether PEMFs can be used to limit the pain and enhance patient recovery after TKA.

**Methods:**

A prospective, randomized, controlled study in 30 patients undergoing TKA was conducted. Patients were randomized into experimental PEMFs or a control group. Patients in the experimental group were instructed to use I-ONE stimulator 4hours/day for 60days. Postoperatively, all patients received the same rehabilitation program. Treatment outcome was assessed using the Knee Society Score, SF-36 Health-Survey and VAS. Patients were evaluated pre-operatively and one, two, six and 12 months after TKA. Joint swelling and Non Steroidal Anti Inflammatory Drug (NSAID) consumption were recorded. Comparisons between the two groups were carried out using a two-tail heteroschedastic Student’s t-test. Analysis of variance for each individual subject during the study was performed using ANOVA for multiple comparisons, applied on each group, and a Dunnet post hoc test. A p value < 0.05 was considered statistically significant.

**Results:**

Pre-operatively, no differences were observed between groups in terms of age, sex, weight, height, Knee-Score, VAS, SF-36 and joint swelling, with the exception of the Functional Score. The Knee-Score, SF-36 and VAS demonstrated significantly positive outcomes in the I-ONE stimulated group compared with the controls at follow-ups. In the I-ONE group, NSAID use was reduced and joint swelling resolution was more rapid than in controls. The effect of I-ONE therapy was maintained after use of the device was discontinued.

**Conclusions:**

The results of the study show early functional recovery in the I-ONE group. I-ONE therapy should be considered after TKA to prevent the inflammatory reaction elicited by surgery, for pain relief and to speed functional recovery.

**Trial registration:**

Current Controlled Trials ISRCTN10526056

## Background

Osteoarthritis (OA) is a degenerative joint disease that affects approximately 10% of adults over the age of 50 [1]. Patients report a progressive increase in pain leading to functional limitations and impaired mobility. OA is the main indication for Total Knee Arthroplasty (TKA). Each year, over 40,000 TKA procedures are performed in Italy [2]. Satisfactory clinical outcome of TKA can depend on several factors including the type of implant and surgical procedure; however, after TKA, local joint swelling, inflammation and pain can delay patient recovery or limit joint function in the long term, and that may ultimately lead to altered posture and reduced mobility [3,4]. A recent study demonstrated that patient post-operative functional recovery after TKA is inversely correlated to the intra-articular concentration of the pro-inflammatory cytokine IL-6, emphasizing the requirement to control the inflammatory reaction of the joint to surgery [5]. In an attempt to favor patient functional recovery after TKA, detailed rehabilitation protocols have been developed including exercise instructions and physiotherapy.

Oral Non Steroidal Anti Inflammatory Drugs (NSAIDs) are used to control pain and inflammation in the operated knee. However, their use for periods exceeding 72 hours in an aged population must take into account the possible negative side effects, such as reduction in renal perfusion and gastric mucosa damage.

Recent studies have indicated that the physiological pathway leading to resolution of inflammatory processes can be mediated by the activation of adenosine receptors, with the A_2A_ adenosine receptors demonstrating the highest anti-inflammatory activity [6,7]. It has been demonstrated that Pulsed ElectroMagnetic Fields (PEMFs) have an agonist activity for the A_2A_ adenosine receptor in neutrophils, chondrocytes and synoviocytes, and this explains the anti-inflammatory effect of PEMFs observed in the knee joint [8,9]. Level I clinical trials conducted in patients undergoing knee joint surgery have demonstrated that PEMF treatment reduces joint swelling, the requirement of NSAIDs to control pain, and the time to functional recovery, and it is well accepted by patients [10,11].

The aim of this study was to test whether treatment with PEMFs, as an adjuvant to standard rehabilitation, could limit pain and joint swelling immediately after TKA and shorten the time to functional recovery.

## Methods

### Patients and design of study

In the period between 2008 and 2010, at the Orthopedic and Traumatology Operative Unit of University of Bari in Italy, a prospective, randomized, controlled study was performed to test whether treatment with PEMFs could favor functional recovery in patients undergoing TKA. The study was designed without placebo devices, following Local Ethical Committee indications that did not consider appropriate to ask patients to devote four hours per day for 60 days to using a placebo device, knowing the anti-inflammatory activity of the treatment. The study was approved by the Local Ethical Committee, and patients signed informed consent for recruitment.

The inclusion criteria were the following: patients aged between 60 and 85 years, presenting with an advanced state of knee OA and scheduled for TKA, with varus or valgus deformity not exceeding 20° or 15°, respectively, and with a flexion contracture of less than 15°. Exclusion criteria were: previous surgery to the same knee, omolateral hip prosthesis, Body Mass Index (BMI; Kg/m^2^) >30, rheumatoid arthritis, autoimmune diseases, systemic diseases, cancer and the use of steroids.

### Biophysical stimulation

Thirty patients were randomly assigned to the experimental and control groups. At selection, the clinician informed the patient regarding the chance to be recruited in a trial with two arms. At enrollment, the patient accepted to be addressed to one of the two groups for the entire duration of the study and the patient signed informed consent for recruitment. The assignment of the patient to experimental or control group was performed using a web-based computer program (http://www.randomization.com) built on the randomization criteria: sex (M/F), age (60-75 years; 75-85 years) and smoking status (yes/no).

Patients in the experimental group were instructed to use PEMFs (I-ONE therapy) four hours per day for 60 days by an independent, unblinded research assistant, who will not be involved in patient care or assessment. Physicians, as well as medical assessors, were blinded to the allocation of patients in the study groups.

**Figure 1:**
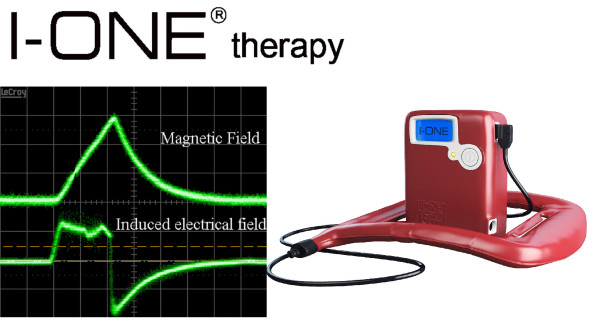
**I-ONE therapy.** Left: waveform of magnetic field 1.5 mT peak value (top); waveform of induced electrical field 0.051 mV/cm peak value, as detected using a standard coil probe (50 turns, 0.5 cm internal diameter of the coil probe, 0.2 mm copper diameter) (bottom). Right: I-ONE PEMF generator.

All devices were provided by the manufacturer at no cost. Treatment began within seven days from TKA, and continued at the rehabilitation centre or at the patient’s home. The battery-operated device (I-ONE, IGEA, Carpi, Italy) generated a peak magnetic field of 1.5 mT at a frequency of 75 Hz (Figure 1); the coil was placed on the operated knee, but not in direct contact with the skin. The apparatus had a timer to record the hours of therapy, allowing patient compliance to be monitored. Patients were instructed to interrupt the treatment if there were adverse events such as a burning sensation or skin irritation. Both groups underwent the same rehabilitation protocol.

### Surgical procedure

For all patients, TKA was performed by the same surgeon and the same anesthesiologist. Anesthesia was obtained using diazepam and selective subarachnoid injection in the space L3-L4 or L4-L5 with 25 GA needles of 2 ml of levobupivacaine at 0.5%. The post-operative pain was treated with continuous venous infusion of tramadol and ketorolac using an elastomeric pump for 48 hours. Using a femoral perineural catheter, 20 ml of levobupivacaine at 0.2% were administered daily, one hour prior to rehabilitation, until the fifth day after surgery. For all patients, from day five until discharge, 1 g of oral paracetamol was administered as required. Cemented Genesis 2 CR total prosthesis (Genesis 2CR, Smith and Nephew, 3175 Coughlin Drive, Memphis, TN 38116, USA) was implanted following a standard procedure.

### Rehabilitation

Kinesitherapy began on the same day of surgery, with passive mobilization on Kinetec (Kinetec RIMEC Fisiotec 2000, Loc Broine 57/a, Roveggio, Bo, Italy) from 60° to 90° bending for 90 minutes. On subsequent days, this was continued with bending from 0° to 90° for 90 min per day using the Kinetec in the afternoon; from day three, patients began cautious passive and active assisted mobilization of hip, knee and ankle, exercises for muscular reinforcement of lower limbs, assisted walking with two crutches and partial weight bearing on the operated limb and isometric exercises for the lower limbs.

### Clinical assessment

Patients were required to complete subjective assessment forms and the physician to complete the objective ones. Assessments were performed pre-operatively and at one, two, six and 12 months post-operatively and included:

the Visual Analogue Scale (VAS), a 10 cm horizontal line, where the left end represents no pain, and the right end maximum possible pain or unbearable pain;

The Knee Society Score including: (*a*) Knee Score to assess pain, range of motion, stability, contracture in bending, active extension deficit and alignment); (*b*) Functional Score to evaluate autonomy in walking, stair climbing, use of a stick or frame. Both score values ranged from 0 to 100 [12].

SF-36 Health Survey that required the patient to answer 36 questions [13]. The mean value of the resulting eight quantitative dimensions was calculated and reported as the final SF-36 score.

Joint swelling score: knee girth was determined by measuring the transverse plane circumference of both knees at midpatellar height in the supine position, using a flexible plastic measuring tape; girth difference in cm between limbs is related to functional outcome after TKA [14]. No girth difference between knees scores 40 (mild), less than 0.5 cm 30 (moderate), between 0.5 and 1 cm 20 (tense), between 1 and 1.5 cm 10 (severe) and more than 1 cm 0 (high).

Patients were asked if they used NSAIDs at each follow-up visit.

### Statistical method

The sample size was calculated on the primary outcome of the study, i.e. the pain, expressed as VAS, given the presence in the literature of several studies concerning the effects of electromagnetic fields on pain [10,11,15].

Starting from two groups that were homogeneous as regards the mean value of VAS at baseline, was hypothesized a difference in the mean VAS value of two units between the two groups from the first month of therapy, with a standard deviation of two units. In the power analysis used to calculate the minimum number of patients per group, the following formula was used:

(1) $$n>2{\left[\frac{\left(z_{2\alpha }+z_{2\beta }\right)\sigma }{\delta }\right]}^{2}$$

Where n is the minimum number of patients per group, $z_{2\alpha }=1.96$ for a two tailed significance of p = 0.05, $z_{2\beta }=0.842$ for a power of 80%, *σ* is the standard deviation and *δ* is the hypothesized difference between groups.

From this, given our assumptions, the minimum number of subjects per group is 15.

The normality distribution of the two samples was tested using the Kolmogorov-Smirnov test and all quantitative variables appeared to be normally distributed. The descriptive analysis of the continuous variables was performed by calculating the mean value and the standard deviation in each group. The two groups were compared at baseline and at all follow-ups by calculating the mean value of the parameters of study and the mean value of the their variations with respect to the baseline. The statistical model employed for comparison of the two groups was the two-tailed heteroschedastic Student’s t-test, whilst for analyses among follow-up and baseline values on each group ANOVA test for multiple comparisons and post hoc Dunnet test was performed.

Contingency tables were used to test differences in categorical variables between the two groups at baseline and at each follow-up visit using the chi square test with Yate’s correction, whilst for analyses on the time effect on each group, non parametric tests for multiple related samples were used with Friedman test.

The statistician was blind regarding the treatment and control group. The statistical analyses were conducted using the Statistical Packages for Social Sciences (SPSS Inc. Chicago, ILL, US).

## Results

At the baseline, no statistically significant differences were observed between the two groups with the exception of the Functional Score, Table 1. Patient compliance was satisfactory, and the average daily use of I-ONE was 3.9 ± 0.5 hours. No side effects were recorded and no patient discontinued the treatment.

**Table 1:** Baseline patient characteristics

	**Control group (#15)**		**I-ONE group (#15)**		**p value**
	**Average,**	**St.Dev**	**Average**	**St.Dev**	
Age (years)	70.5	8.1	70.0	10.6	0.894
Weight (kg)	73.4	13.3	77.6	16.1	0.464
Height (cm)	163.0	7.9	164.9	7.4	0.502
Knee Score	42.1	13.9	39.5	13.8	0.620
Functional Score	21.0	18.8	39.3	25.1	0.032
SF-36 Health Survey Score	28.6	8.1	36.7	13.7	0.060
VAS	7.6	1.8	6.5	2.0	0.107
Joint Swelling Score	18.0	12.6	19.3	10.3	0.754

Characteristics of the groups at baseline: control group and I-ONE therapy group.

### Knee score

**Figure 2:**
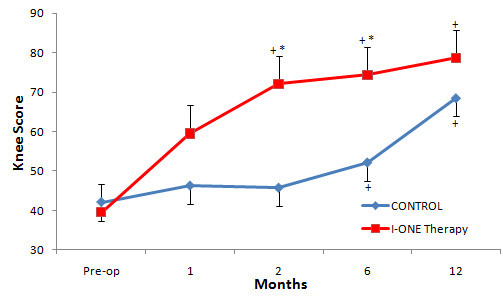
**Knee Score.** Mean values of Knee Score in the control group and I-ONE therapy group during the study. Vertical bars represent the standard error. *p < 0.0001, p values refer to a comparison between groups at each follow-up visit by two tailed heteroschedastic Student’s t-test. ^+^p < 0.05, statistically significant difference versus pre-op.

In both groups, follow-up Knee scores were greater than the pre-operative baseline values, and the changes were statistically significant at two, six and 12 months (p < 0.05) in the experimental group, but only at six and 12 months in the control group (p < 0.05). Intergroup analysis demonstrated that the Knee score in the I-ONE group was significantly higher after two months (72.1 ± 15.3 vs. 45.7 ± 17.1; p < 0.0001) and six months (74.4 ± 14.5 vs. 52.1 ± 15.3; p < 0.0001) than in controls. At 12 months, no difference was observed between groups (p = 0.097), (Figure 2).

### Functional score

**Figure 3:**
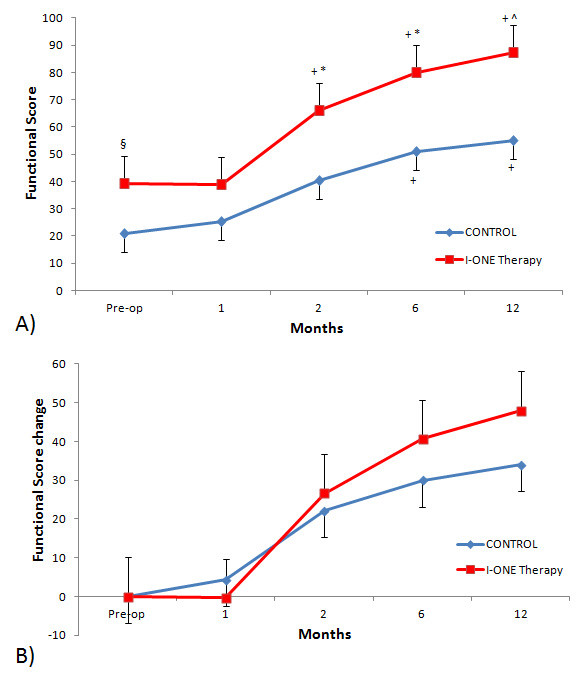
**Functional Score.(A)** Mean values of Functional Score in the control group and I-ONE therapy group during the study. Vertical bars represent the standard error. *p < 0.0001, ^p < 0.005, ^§^p < 0.05, p values refer to a comparison between groups at each follow-up visit by two tailed heteroschedastic Student’s t-test. ^+^p < 0.05, statistically significant difference versus pre-op. **(B)** Mean changes in Functional Score in control and I-ONE groups during the follow-up compared with baseline values. Vertical bars represent standard errors.

The Functional Score increased with respect to baseline and was significant at two months follow-up (p < 0.05) in the experimental group, whilst in the control group the increase became significant at six months. There was no statistically significant difference between the two groups one month after surgery (p = 0.124), but there were highly significant differences between the groups at two months (66.0 ± 28.7 vs. 40.4 ± 17.5, p < 0.0001), six months (80.0 ± 19.4 vs. 51.0 ± 18.2, p < 0.0001) and 12 months (87.3 ± 16.8 vs. 55.0 ± 33.2, p < 0.005) (Figure 3A).

The baseline values of Functional Score in the I-ONE group were significantly higher than those of the control group. Therefore, a further analysis was conducted, taking into account the changes recorded at follow-up visits versus the baseline. This analysis did not demonstrate significant differences between groups at follow-ups; however, at six and 12 months, the experimental group values were higher than controls (Figure 3B).

### SF36 health survey

**Figure 4:**
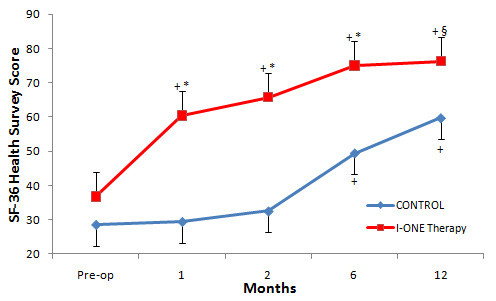
**SF-36 Health Survey Score.** Mean values of SF-36 Health Survey in the control group and I-ONE therapy group during the study. Vertical bars represent the standard error. *p < 0.0001, ^§^p < 0.05, p values refer to a comparison between groups at each follow-up visit using two tailed heteroschedastic Student’s t-test. ^+^p < 0.05, statistically significant difference versus pre-op.

One month after TKA the SF-36 Health Survey score in the I-ONE group was significantly higher than the control group (60.5 ± 14.4 vs. 29.5 ± 10.5, p < 0.0001) and the baseline (p < 0.05). The difference between groups was maintained at all follow-up visits: two months (65.8 ± 15.2 vs. 32.5 ± 9.2, p < 0.0001), six months (75.1 ± 9.6 vs. 49.5 ± 17.2, p < 0.0001) and 12 months (76.3 ± 8.7 vs. 59.7 ± 19.6, p < 0.05) (Figure 4).

### VAS

**Figure 5:**
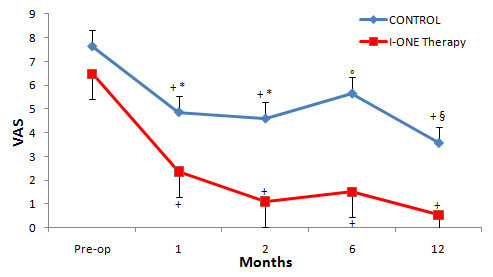
**VAS Score.** Mean values of VAS in the control group and I-ONE therapy group during the study. Vertical bars represent the standard error. *p < 0.0001, °p < 0.001, ^§^p < 0.05, p values refer to a comparison between groups at each follow-up visit using two tailed heteroschedastic Student’s t-test. ^+^p < 0.05, statistically significant difference versus pre-op.

The pre-operative pain values of both groups were high (I-ONE group 6.5 ± 2.0 vs. control 7.6 ± 1.8, p = 0.107). Figure 5 demonstrates the decline in VAS values during follow-up visits; ANOVA test for repeated measurements shows significantly lower values than baseline in both groups (p < 0.05). VAS values were significantly lower in the experimental than the control group at all follow-up visits: one month (2.4 ± 1.6 vs. 4.9 ± 1.8, p < 0.0001), two months (1.1 ± 1.0 vs. 4.6 ± 1.8, p < 0.0001), six months (1.5 ± 2.8 vs. 5.6 ± 2.9, p < 0.001) and 12 months (0.5 ± 1.3 vs. 3.6 ± 3.9, p < 0.05).

### Swelling

Swelling was resolved in both groups and was significantly reduced at the follow-up visits with respect to baseline (p < 0.05). The percentage of subjects with mild to moderate swelling was significantly higher in the I-ONE group with respect to control group at 1 and 2 months after surgery (p < 0.05 and p < 0.0001, respectively). No difference between groups was observed at 6 and 12 months (Table 2).

**Table 2:** Joint swelling

	**Control group**	**I-ONE group**	**p value**
Pre-op	27	20	ns
1 month	67	100	<0.05
2 months	33	100	<0.0001
6 months	87	100	ns
12 months	87	100	ns

Percentage of patients with mild to moderate swelling in control and I-ONE treated groups at each study visit. p values refer to a comparison between groups at each follow-up visit using chi square test with Yate’s correction.

### Use of NSAIDs

One month after TKA, 33% of patients in the I-ONE group used NSAIDs as compared with 93% in the control group (RR = 8.11 C.I. 1.23-53.57, p = 0.0017). At two and six months, the percentages of patients using NSAIDs in the experimental and control group were 7% and 86% (RR = 7.39 C.I. 2-27.26, p < 0.0001), and 7% and 46% (RR = 2.57 C.I. 1.31-5.06, p < 0.05), respectively. At 12 months, no difference was observed between groups.

## Discussion

TKA is the most common and effective surgical procedure for the treatment of OA, leading to satisfactory functional recovery in patients. However, it can be associated with moderate or severe post-operative pain and an intense inflammatory reaction. Pain stems from the onset of loco-regional inflammation, and the presence of pro-inflammatory cytokines (interleukin-1beta: IL-1β, IL-6), tumor necrosis factor-α, histamine, bradykinin, prostaglandin, serotonin, substance P and acetylcholine, which stimulate nociceptors and induce hyperalgesia and allodynia [16-18]. It has been demonstrated that the increase in SF-36 score is slow in the 1^st^ post-operative month and then accelerates at six and 12 months, when local inflammation and pain have been resolved [19,20]. Inflammation and pain at the joint after TKA can limit rehabilitation and delay functional recovery [18].

Innovative therapeutic strategies are required to locally control the inflammatory reaction following TKA.

It has been demonstrated that PEMFs have an agonist effect on A_2A_ adenosine receptors, and this explains the anti-inflammatory effects observed in experimental [8,9,21] and clinical [10,11] studies. Knees in sheep undergoing osteochondral grafts exposed to PEMFs show a reduced concentration in the synovial fluid of pro-inflammatory cytokines (IL-1β, tumor necrosis factor alpha: TNF-α) [22]. *In vitro*, PEMFs exposure prevents the release of PGE_2_ by synoviocytes cultured in the presence of pro-inflammatory cytokines (lipopolysaccharide, TNF-α) [23].

These results support the rational basis for the use of PEMFs to control the inflammatory reaction that follows surgical procedures. Two randomized, prospective and double-blind studies have been conducted in patients undergoing arthroscopic procedure on the knee. The first study included patients with cartilage lesions undergoing microfractures, while the second concerned patients undergoing anterior cruciate ligament reconstruction. In both studies, early functional recovery of the joint and diminished consumption of NSAIDs were reported [10,11].

In patients undergoing hip revision surgery, PEMFs treatment resulted in early pain control and enhanced functional recovery [15]. Straburzynska-Lupa et al., in a clinical study of 25 patients treated with PEMFs combined with local cryotherapy following TKA, describe reduced pain, reabsorption of the edema and improved functional recovery [24].

On the basis of the above evidence, this prospective, randomized and controlled trial was conducted to verify whether the treatment with I-ONE therapy, in addition to standard rehabilitation, could control pain, reduce swelling and improve functional recovery in patients undergoing TKA.

The Knee Score, based on objective examination of joint, was significantly better for the I-ONE group at two and six months after TKA, indicating that the positive effect is maintained even after the end of the treatment. Furthermore, the values of the Knee Score in the control group at 12 months were comparable with those observed in the experimental group two months post TKA.

The Functional Score, which measures the subjective evaluation of functional recovery, does not demonstrate statistically significant differences between the treated and control groups. These results are in agreement with those reported by other authors concerning the lack of correlation between the parameters of the subjective evaluation scales and those of the clinical-functional scales [11,25].

SF-36 Health Survey score demonstrates that patients treated with I-ONE therapy after TKA had significantly higher values than the control group until month twelve. Furthermore, at 12 months the SF-36 value of the control group equated with that of the I-ONE group at one month.

The values observed for the control group were compared with information available in the literature for Knee Score, Functional Score and SF-36 Health Survey. Breugem et al. [26] reported a change in Knee Score of 30 points over 12 months, which compares favorably with the 26 points change in this study. The major difference was observed for Functional Score, where the changes were 24 points versus 34 in the present study. In the control group, the SF-36 value doubled at 12 months follow-up and similar findings have been reported by Brandes et al. [27]. In this study, the score values in the control group were comparable to those in other studies and the differences observed can be ascribed to the populations investigated, and surgical and rehabilitation procedures [28,29].

Pain, monitored on the VAS scale, at all follow-ups was significantly lower in treated group with respect to control one. Swelling was resolved earlier in the treated group; no significant difference between groups was observed at 6 and 12 months. Other authors have reported that limitation due to swelling was important during the acute period (one month after TKA) and the improvement continued until 12 months after surgery [14,30]. At one month after TKA, only 33% of patients in the experimental group, versus 93% in the control one, required NSAIDs (p = 0.0017). The above observations are in agreement with previous reports concerning patients undergoing knee arthroscopic surgery [10,11]. At six months, the number of patients using NSAIDs was still high (46%) in the control group (p < 0.05 vs experimental group); however, it further diminished and at 12 months there was no difference between groups. It is accepted that recovery after TKA is longer than after total hip prosthesis. Jones et al., using a large series of patients (#256) undergoing TKA, reported pre-operatively average pain score of 43, increasing to 75 six months after surgery (100 indicating no pain); the study did not report the use of NSAIDs [31]. Baker et al. reported that 19% of patients still suffered persistent pain one year after TKA [32].

The lack of a placebo group is a limitation of this study, but it must be acknowledged that all clinical evaluations were carried out by physicians unaware whether the patient belonged to the control or experimental group. The small population size did not allow to reliably apply the group x time interaction effect test in the analysis of the results. Furthermore, the limited number of patients may explain the difference in Functional Scores observed at the baseline between the two groups. Patients’ compliance was a concern, as the use of I-ONE for four hours per day for 60 days requires significant commitment. However, the treatment was well accepted, and patient compliance was high (3.9 hours per day average use) as the device is portable, battery operated and can be worn while walking or at rest.

## Conclusions

This study shows that I-ONE therapy can be started soon after TKA. The treatment has been well tolerated and no negative side effects have been reported. The lack of the placebo group prevents to precisely quantify the contribution of the treatment to the observed pain relief and shortening of functional recovery. Nevertheless, I-ONE therapy may represent an important adjunct to postoperative treatment by preventing the detrimental effect of inflammation elicited by TKA on joint tissues, with short and long term positive benefits for patients.

## Competing interests

SS, FDT are employees of IGEA SpA – Clinical Biophysics

The authors BM, AN, LM, VPA and VPE declare that they have no competing interests.

## Authors’ contributions

BM, AN and SS participated in the study design. AN and SS designed the protocol and helped with data interpretation. BM, AN, LM, VPA and VPE carried out the study. SS, FTD and AN interpreted the results and drafted the manuscript. FDT performed the statistical analysis. All authors read and approved the final manuscript.

## Pre-publication history

The pre-publication history for this paper can be accessed here:

http://www.biomedcentral.com/1471-2474/13/88/prepub
